# Bioinformatic detection of copy number variation in *HNF4A* causing maturity onset diabetes of the young

**DOI:** 10.1111/cge.13599

**Published:** 2019-07-15

**Authors:** Amanda J. Berberich, Arati Mokashi, Adam D. McIntyre, John F. Robinson, Henian Cao, Jian Wang, Robert A. Hegele

**Affiliations:** ^1^ Department of Medicine and Robarts Research Institute, Schulich School of Medicine and Dentistry Western University London N6A 5B7 Canada; ^2^ Division of Endocrinology, Department of Pediatrics IWK Health Centre, Dalhousie University, Faculty of Medicine Halifax B3K 6R8 Canada

## Abstract

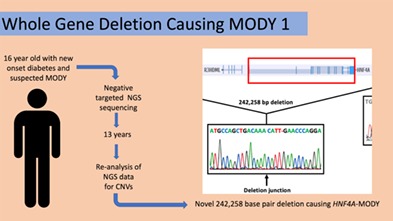

1

*To the Editor*:

Maturity onset diabetes of the young (MODY)^1^, ^2^ is currently under‐recognized clinically,^3^ and may also be under‐recognized by molecular genetic analysis.^1^ Genetic testing for MODY is presently accomplished primarily using next generation sequencing (NGS) techniques.^1^, ^2^, ^4^ However, these techniques are historically unable to detect copy number variations (CNVs), defined as large‐scale deletions or duplications in genomic DNA. We report here on a large‐scale heterozygous CNV in *HNF4A* causing MODY in an individual who initially tested negative for mutations by DNA sequencing alone.

The proband carrying this mutation was a Caucasian female, diagnosed with diabetes at age 16. She has a normal body mass index with negative diabetes‐associated autoantibodies and a significant family history of diabetes. She was managed effectively with a sulphonylurea for several years, with improvement in HbA1c from 7.5% to 5.6% compared to multiple daily injections of insulin. Sulphonylurea therapy eventually became ineffective and insulin was re‐initiated.

The proband provided informed consent and DNA was obtained from whole blood in 2005 under a protocol approved by the University of Western Ontario Ethics Review Board (#07920E). LipidSeq,^5^ a targeted NGS and bioinformatics platform, was used to assess for pathologic mutations in known MODY‐associated genes, and none were detected. Her original NGS output data was recently re‐analyzed 13 years after the original testing was completed using the VS‐CNV caller function in VarSeq v1.4.3 (Golden Helix, Bozeman, Montana), a technique that takes advantage of depth‐of‐coverage (DOC) information provided from raw NGS data to predict the presence of CNVs. A whole‐gene deletion of *HNF4A* (MODY1) was detected (Figure 1B). The CNV average ratio for this deletion was 0.531123 with a Z‐score of −6.6691 (significance threshold −5) for the targeted NGS data and an average ratio of 0.577031 with a Z‐score of −2.94078 (significance threshold −2) and a *P*‐value of <1 × 10^−30^ for confirmatory whole exome sequencing (WES). This deletion has not been previously reported and would be expected to be causal for MODY.

**Figure 1:**
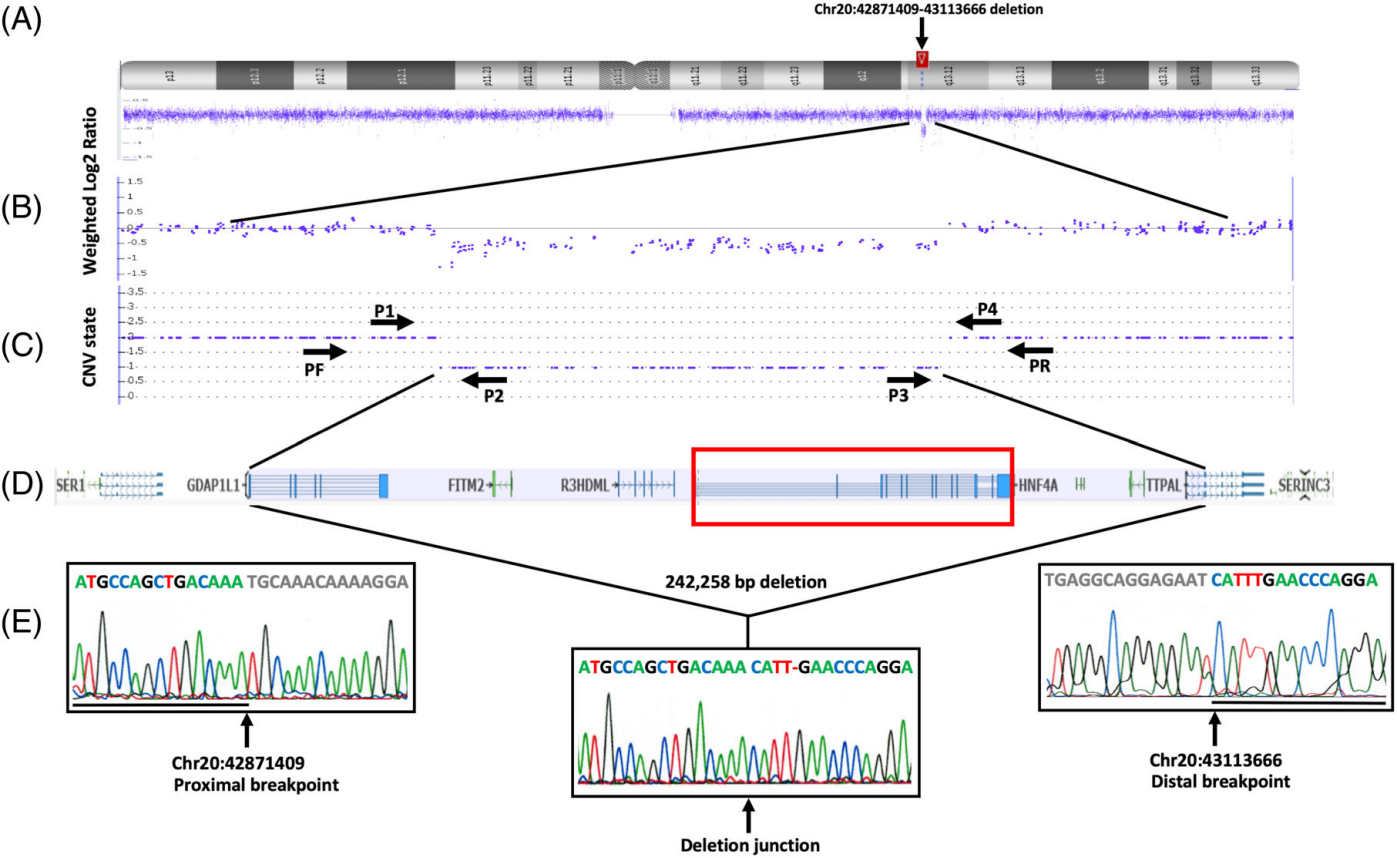
Confirmation of copy number variation (CNV) detection using NGS output data in *HNF4A*. A, CytoScan genotyping output showing loss of zygosity on chromosome 20q13, with reduced intensity of probe signals, expanded in panel B. C, Corresponding region with the same deletion detected using depth‐of‐coverage analysis from next generation sequencing (NGS) and whole exome sequencing (WES) output data. D,The genes that map within the deleted region, including the entire *HNF4A* gene. E, Sanger sequencing electropherogram tracings that demonstrate normal DNA sequences in the vicinity of the proximal 3′ (left side) and distal 5′ (right side) breakpoints of the deletion. Missing internal sequence from the deleted allele is shaded gray. In the bottom center is the Sanger sequencing electropherogram tracing from the proband, showing the deletion junction, with absence of the intervening 242 258 nucleotides. Amplification primer locations are indicated in panel C. Primer design and reaction conditions are available upon request [Colour figure can be viewed at http://wileyonlinelibrary.com]

The detected CNV was further confirmed using CytoScan array (Affymetrix Santa Clara, California), a clinically validated tool to detect CNVs, which similarly demonstrated a large‐scale deletion spanning this region (Figure 1A). The breakpoint was established using polymerase chain reaction (PCR)‐based probe analysis and Sanger sequencing using primers designed to target either side of the suspected breakpoint. This confirmed the presence of a heterozygous 242 258 bp deletion spanning chr20:42871409‐43 113 666 (Figures 1C‐E).

A diagnosis of MODY1, seen here, leads to a mild‐moderately severe, progressive form of diabetes.^3^ As demonstrated by the proband, MODY1 may be optimally treated with low‐dose oral sulfonylurea therapy for many years, although insulin therapy may eventually be required.^4^ Our findings suggest that incorporating CNV analysis routinely during genetic testing for MODY may improve diagnostic yield and may also help establish the true prevalence of CNVs as a cause of MODY.

## Data Availability

There is no associated shared data.
